# Supplementary material for: A statistical framework for comparing epidemic forests
Source: PLoS Comput Biol. 2026 Jun 1;22(6):e1014271. doi: 10.1371/journal.pcbi.1014271 (PMC13225645; doi:10.1371/journal.pcbi.1014271)
Supplement: S1 File — (PDF) [file pcbi.1014271.s001.pdf]

Simulation Framework

Our simulation study followed a two-step process designed to evaluate how effectively the proposed statistical methods could distinguish between epidemic forests derived from distinct generative processes. First, we simulated *reference* transmission trees using a branching process model with varying offspring distributions. Second, we generated epidemic forests from these reference trees by re-assigning infector-infectee relationships from a new offspring distribution, conditional on the reference's tree case identifiers and dates of infection producing collections of plausible transmission trees that reflect the underlying dynamics of each scenario. This approach provided a controlled environment with known ground truth against which we could systematically assess the discriminatory power of our statistical tests across different epidemic sizes, forest sizes, and offspring distribution parameters.

Branching process model

Our simulations employ the discrete-time stochastic branching process implemented in *simulacr* (`https://github.com/CyGei/simulacr`). Each simulation was initiated with a single infected individual (the index case), from whom infections propagated across successive generations based on specified offspring and generation time distributions.

simulacr tracks the propagation of infections through successive generations by computing the force of infection (FOI) at each time step $[t, t+1)$. For each infected individual i, we define t_i as their infection time and R_i as their case reproduction number—the expected number of secondary cases they generate in a fully susceptible population. The generation time—the interval between the infection of a primary case and the infection of its secondary cases—follows a probability mass function $g(t)$, where $g(t) = 0$ for $t \leq 0$. At each time step, the model sums the infectious contribution from all active cases to determine the overall force of infection, then probabilistically generates new infections from the susceptible population ($S(t)$) and assigns their respective infectors according to their relative contribution to transmission.

The FOI generated by case i at time t is defined as:

$$\lambda_i(t) = R_i\, g(t - t_i) \tag{1}$$

The total FOI at time t arising from all infectious individuals at this time (denoted by the set $I(t)$) is given by:

$$\Lambda(t) = \sum_{i \in I(t)} \lambda_i(t) \tag{2}$$

Each susceptible individual j (with $j \in S(t)$) faces an infection probability during the interval $[t, t+1)$ of:

$$p_j = 1 - e^{-\Lambda(t)} \tag{3}$$

Once a susceptible individual becomes infected at time $t+1$, a specific infector is drawn from a multinomial distribution where the probability that case $i \in I(t)$ is the infector is defined as:

$$P(i \mid \text{infection at } t+1) = \frac{\lambda_i(t)}{\Lambda(t)} \tag{4}$$

To achieve exactly ε cases per outbreak, we initialised simulations with 10,000 susceptible individuals and terminated them upon reaching exactly ε infections, thereby excluding saturation effects. This truncation scheme led to right-censoring, where cases infected within one generation time of the truncation date likely had not realised their expected number of secondary infections R_i.

For all simulations, we modelled the generation time using a discretised probability mass function (PMF) with a mean of 12 days and a standard deviation of 6 days to enable considerable entropy across reconstructed forests (supplementary S5 Fig).

Each simulated transmission tree returns the case identifiers of infected individuals and their infection dates which will be used for forest generation.

Forest generation

For each simulated reference transmission tree $\mathcal{T}^{\varepsilon}_{(R_0,k)}$, we generated epidemic forests $\mathcal{F}_{\mathcal{T}^{\varepsilon}_{(R_0,k)}}$ by reassigning infector-infectee relationships given a new offspring distribution $\text{NegBin}(R_0', k')$ while conditioning on the reference tree's set of infections $\mathcal{I}_{\mathcal{T}^{\varepsilon}_{(R_0,k)}} = \{(v, t_v)\}_{v=1}^{\varepsilon}$, where v denotes case identifiers and t_v their infection times. Each forest comprised of $m = 200$ trees.
For each tree in the forest, we followed a three-step process:

- **Offspring sampling:** we sampled $R_i \sim \text{NegBin}(R_0', k')$ for all ε cases, where R_i denotes the number of secondary infections generated by case i. To ensure that the epidemic sets off, we constrained the index (root) case to have $R_1 \geq 1$ by sampling from a truncated negative binomial distribution when $R_1 < 1$.

- **Force of infection (FOI) calculation:** For each case i, with infection time t_i and reproduction number R_i, we computed the FOI exerted by i at time t as:
$$\lambda_i(t) = R_i g(t - t_i)$$
where $g()$ is the generation time probability mass function.

- **Ancestry assignment:** cases were assigned infectors sequentially in order of their infection times. For each case j, infected at time t_j, we identified the set of ancestors $\mathcal{A}(t_j) = i : t_i < t_j$ and selected the infector by sampling from the multinomial distribution with probability relative

to the contribution of i on the whole FOI at time t_j:

$$P(i \to j) = \frac{\lambda_i(t_j)}{\sum_{i' \in \mathcal{A}(t_j)} \lambda_{i'}(t_j)}$$

This procedure ensures that each generated tree $\mathcal{T}_k = (V, E_k)$ in forest $F_{\mathcal{T}}(R_0', k')$ shares the same vertex set V and infections times t_v as the reference tree $\mathcal{T}$, but with edges resampled according to the FOI generated under the new offspring distribution $\text{NegBin}(R_0', k')$. An epidemic forest is thus a collection of transmission trees that share the same set of infected individuals but different ancestral relationships, all consistent with a given offspring distribution.

Validation of offspring distribution

To assess whether the forest generation procedure preserves the target offspring distribution, we conducted a simulation study over a grid of reproduction numbers $R \in 1.5, 2, 3$ and dispersion parameters $k \in 0.1, 0.5, 3$. For each (R, k) pair, we simulated 50 reference transmission trees with $\varepsilon = 200$ cases over 365 days. The number of secondary infections per case was drawn from a $\text{NegBin}(R, k)$ distribution, and infection times from a discretised gamma generation time distribution (mean 12 days, sd 6 days).

Trees were truncated at $\varepsilon = 200$. Cases infected near the end of the observation period may not realise all secondary infections, leading to right-censoring. To account for this, any case infected within one generation time of the last observed infection was flagged as censored, where one generation time is defined as the 99th percentile of the generation time cumulative distribution function. Censored cases were retained as offspring when counting secondary infections of uncensored parents, but excluded as potential parents. This avoids downward bias due to incomplete follow-up.

The empirical offspring distribution among uncensored parents closely matches the theoretical $\text{NegBin}(R, k)$ probability mass function across all parameter settings (S8 Fig), confirming that the simulation and censoring procedure recover the target distribution. Right-censoring reduces the number of cases available for estimation, particularly at higher R, where faster growth concentrates infections near the end of the observation window (S9 Fig).

To evaluate the forest generation procedure, we generated for each reference tree a forest of 50 trees by reassigning infector–infectee relationships. For each tree in the forest, we counted the number of secondary infections per uncensored case and compared the resulting offspring distribution to that of the reference tree using quantile–quantile (QQ) plots (S10 Fig). At evenly spaced probability levels, we computed offspring quantiles for both the reference tree and each tree in the forest and plotted them against each other. Across all (R, k) combinations, the density is concentrated along the identity line, indicating good agreement between the two distributions. To check for systematic bias, we also computed the difference between matched quantiles at each probability level

(S11 Fig). Median deviations are centred at zero across all settings, confirming the absence of bias. Variance increases at higher quantiles due to greater sampling variability in the tail of the negative binomial, particularly under strong overdispersion ($k = 0.1$). Together, these results show that the forest generation procedure preserves the offspring distribution.

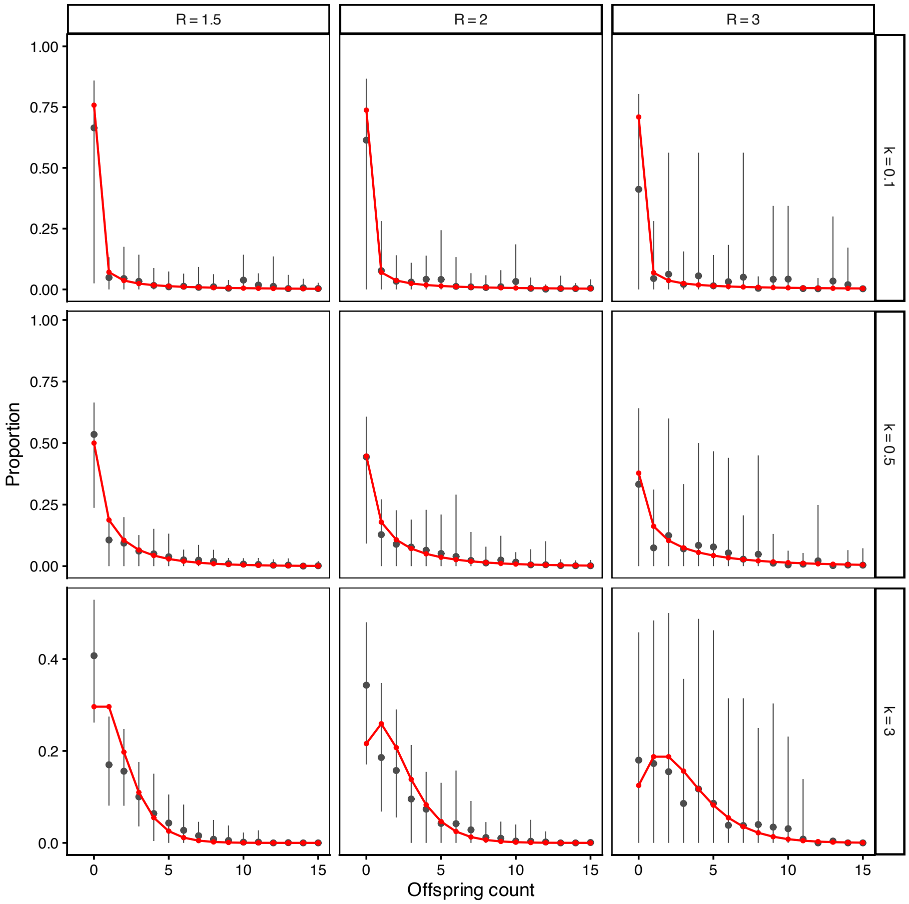

Figure S8: **Empirical versus theoretical offspring distribution.** For each (R, k) combination, grey points and error bars show the mean proportion of cases with a given number of secondary infections and corresponding 95% empirical quantile intervals across 50 reference trees after right-censoring. The red line shows the theoretical $\mathrm{NegBin}(R, k)$ probability mass function. The empirical distribution closely tracks the theoretical expectation, with wider intervals at higher R, where fewer uncensored cases are available, and at lower k, where overdispersion increases sampling variability.

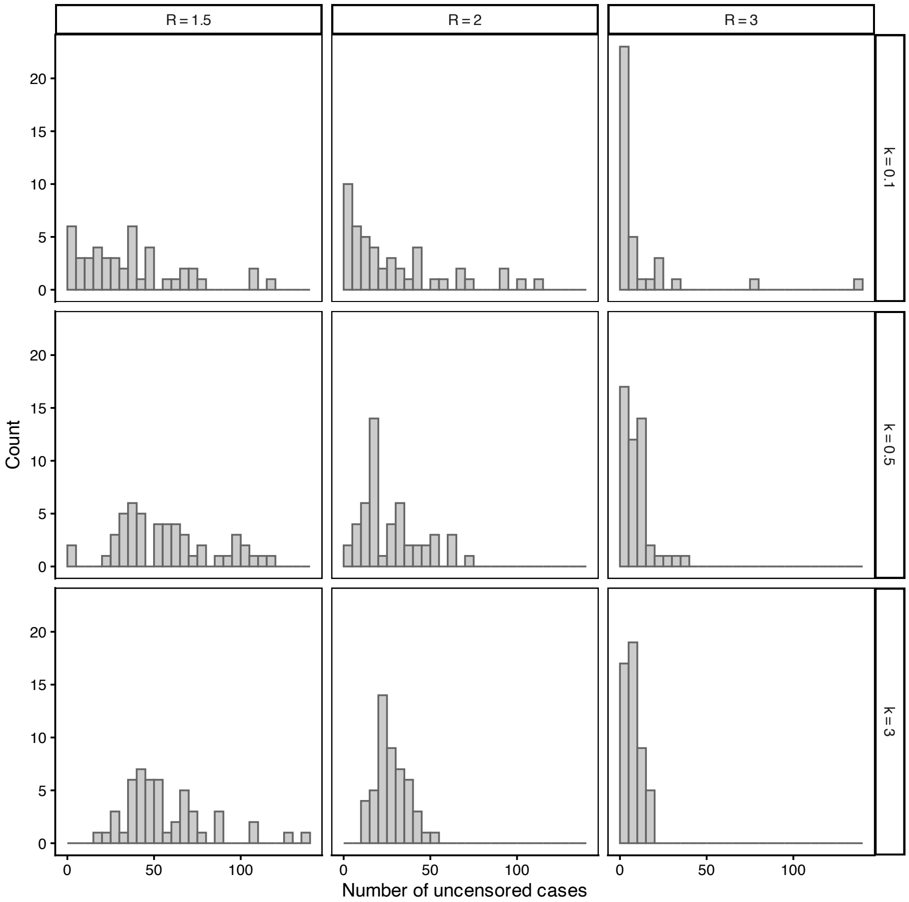

Figure S9: **Effect of right-censoring on effective sample size.** Histograms of the number of uncensored cases per reference tree across all (R, k) settings. Higher R values produce faster-growing epidemics that concentrate more cases near the end of the observation window, resulting in fewer uncensored parents available for offspring estimation.

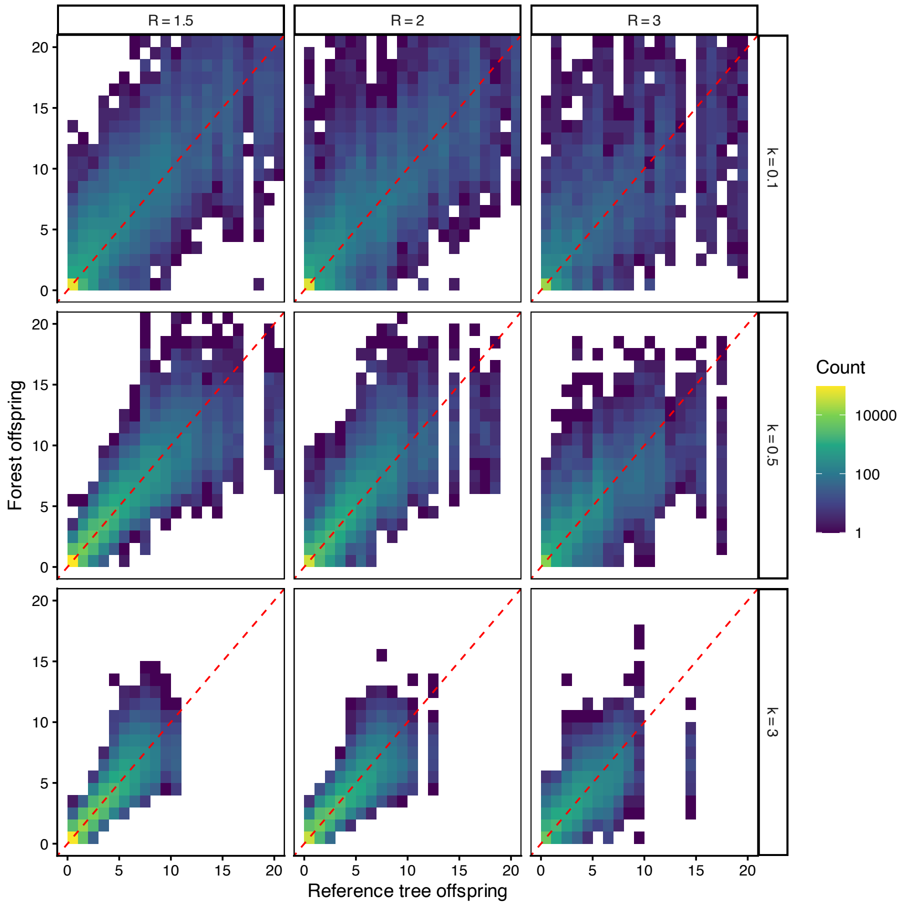

Figure S10: **Quantile–quantile comparison of offspring distributions between reference trees and epidemic forests.** For each reference tree and each of the 50 trees in its forest, we computed offspring quantiles at the same evenly spaced probability levels and paired them. The plot bins these pairs into a grid of unit squares and colours each square by the number of pairs it contains (on log_{10} scale). If the two distributions agree, pairs fall along the $y = x$ line (dashed red). Across all (R, k) combinations, the density is concentrated along the diagonal, confirming that the forest generation procedure preserves the offspring distribution (see also S11 Fig).

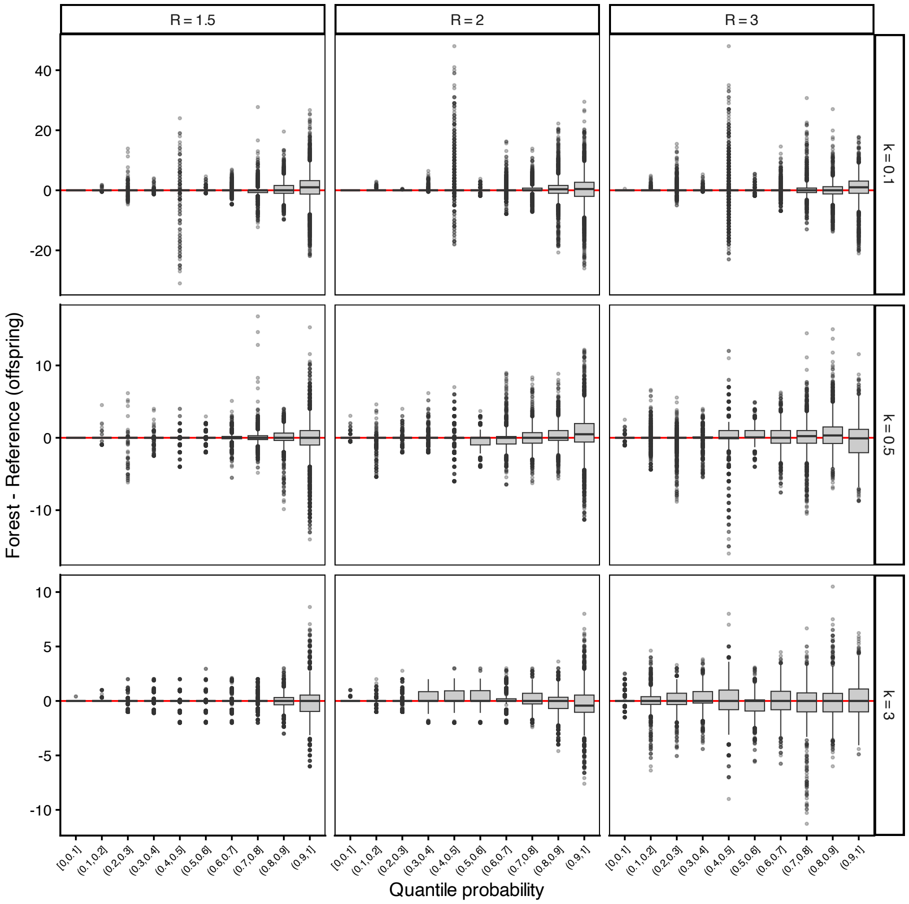

Figure S11: **Deviation between forest and reference quantiles of secondary infections.** Difference between forest and reference quantiles, grouped by quantile probability. The red line marks zero deviation. Boxplots are centred at zero across all bins, indicating no systematic bias. Increased variance at higher quantiles reflects greater sampling variability in the tail of the negative binomial distribution, particularly under strong overdispersion ($k = 0.1$).
