# Supplementary material for: A statistical framework for comparing epidemic forests
Source: PLoS Comput Biol. 2026 Jun 1;22(6):e1014271. doi: 10.1371/journal.pcbi.1014271 (PMC13225645; doi:10.1371/journal.pcbi.1014271)
Supplement: Attachment — Submitted filename: response to reviewers.docx [file pcbi.1014271.s011.docx]

Response to Reviewers

PLOS Computational Biology (PCOMPBIOL-D-25-00809) - [EMID:dd2b47f2aa6ae8d5]

Email of 22/06/2025

# Reviewer 1:

The paper proposes statistical methods for the comparison of large groups of epidemic transmission trees, using either a chi-squared test or a non-parametric version of a multivariate analysis of variance test (PERMANOVA). This is a useful contribution to the field and definitely has a potential home in the literature. The paper is concise and clear. I have a major concern about the approach which needs addressing, and I also think that further simulations are strongly warranted.

What really concerns me is whether the difference in degree distribution is what drives the dissimilarity of the forests at all. The vertices here are labelled, and both methods use the labels. The chi-squared version has the usual contingency table, and PERMANOVA uses an ordered vector of distances. Each node in one set of forests must have a twin in the other. How are these twins assigned? It seems to me that the answer is “arbitrarily”. Unless this is poorly explained, each comparison is made between forests all of which have the same underlying true tree (p14). If this is not true and members of the same forest can have a different true tree, then my concern goes away. But if not, if nodes i and j in one tree are a transmission pair in the tree underlying forest A, they may be distantly related in the one generating forest B. The vector or matrix is going to be very different even if the underlying mathematical model is the same. In this case, does this test anything other than that if true trees are different? If not, why change the generating model at all?

That aside, the other problem I have with this presentation is that the simulation exercise is much too rudimentary and only scratches the surface of the possible applications. It proposes many of those applications without exploring them, and since this is not a long piece of work in its current iteration, I think there is considerable scope for that exploration.

For example:

• The issue of unsampled cases is raised briefly on page 9 but not explored. Could you see how well this works to identify if two sets of trees come from epidemics of different sizes but with the same number of observed nodes?

We agree that this is an important direction for further work. Our current analysis focuses on comparing outbreaks with the same number of sampled cases, and addressing differences in underlying epidemic size would require additional simulation and evaluation that are beyond the scope of this paper. We intend to explore this in future work, together with additional metrics that can assess differences driven by unsampled infections.

• You stop the simulation at 100 days (p20). Another use case would be to see if this can discriminate between finished and ongoing epidemics with the same number of observed nodes.

We now truncate all transmission trees at the exact target epidemic size, so none of the revised simulations represent finished epidemics. We agree that assessing whether the method can discriminate between finished and ongoing epidemics with the same number of observed nodes would be a valuable extension. This analysis would require additional simulation work that lies beyond the scope of the present study, but we plan to consider it in future work.

• The number of different generating models you could compare is effectively infinite, but using one comparison only does feel inadequate.

The simulation study now spans a broad range of generative models, evaluating each test’s performance across combinations of R0 (1.5, 2, 3) and k (0.1, 0.3, 0.5, 1, Poisson like), resulting in a total of 720,000 forest comparisons.

Minor points:

Please put line numbers in a revised version.

Apologies, this has now been added.

Page 2:

Paragraph 2: Another reason for uncertainty (perhaps the key one) is the lack of discriminatory power in choosing between possible transmission pairs (the subject of the Campbell et paper in the genomics realm).
Thank you, we added this point to the paragraph [line 72].

Paragraph 3: “…determining whether differences between them represent meaningful variations in transmission dynamics or are due to sampling and model uncertainty is challenging.” Do you mean MCMC sampling or epidemic sampling? If the latter, I do not think this paper shows how its method helps.
We meant MCMC sampling, we clarified the sentence in the revised manuscript [line 84].

Paragraph 4: MCMC diagnostics are not used to compare competing approaches; if the diagnostics suggest a problem, the analysis has failed before you get to the comparison. It’s not clear what “model reliability” means here but the same comment could also apply.
We agree that MCMC diagnostics do not compare competing approaches directly. In the revised text, we clarified that these diagnostics assess the reliability of the inference procedure itself, rather than the inferred trees, while consensus trees are used to summarise the posterior samples [line 93].

Page 3:

Paragraph 1: I do not see how consensus trees are “abstract representations” any more than actual transmission trees are, and if your consensus tree has loops, it is clearly produced using a bad algorithm. The maximum parent credibility and Edmonds trees are used in the papers that are cited here!

We have clarified in the revised manuscript that by *consensus tree* we simply mean the tree formed by selecting, for each case, the infector with the highest posterior support. While we agree that the Edmonds algorithm is an appropriate way to enforce a valid transmission tree topology by removing cycles and ensuring at most one infector per case, we also note that the resulting consensus Edmonds tree may correspond to a combination of ancestries that was never observed as a complete tree in the posterior. This is because it combines high-support edges that may be mutually incompatible in the joint posterior distribution. We now make this limitation explicit in the revised text [line 106].

Paragraph 3: Why is it important that PERMANOVA is multivariate when the use case here is univariate?

The PERMANOVA is here applied to collections of trees, which are themselves embedded in multivariate spaces (Holmes 2003, Jombart et al. 2017). In the case of transmission trees with ‘*n*’ cases, for instance, each tree is represented by a matrix of pairwise distances between cases, so usually embedded in an *n*-dimensional space.
Holmes, 2003: <https://pages.stat.wisc.edu/~larget/phylogeny/holmes-thPopBio-2003.pdf>
Jombart et al, 2017: <https://pubmed.ncbi.nlm.nih.gov/28374552/>

Page 5:

Paragraph 1: 200 cases is quite small given how fast the method seems to work; I would like to see this extended into the thousands.
We thank the reviewer for this suggestion. While our comparison method is relatively fast, outbreak reconstruction methods typically require several hours, even for 200 cases. In practice, larger epidemics are rarely densely sampled enough for reliable reconstruction, so the epidemic sizes considered in our simulations are representative of realistic applications.

Page 9:

Paragraph 1: Could you not handle multiple introductions in PERMANOVA by very heavily weighting the distance between the root nodes of each introduction?
It is not clear how figure 1 illustrates this dedicated category.
Developing suitable distance metrics for transmission trees is an exciting direction with many possible approaches, and we feel this would be more appropriate as a separate paper. We agree that Figure 1 does not explicitly depict this case, which is not considered in our study, and have removed the reference to the figure from this sentence.

Is using different variables as edge weights in the same analysis how you could make use of PERMANOVA being multivariate?
Using different variables as edge weights would indeed be a potential application of our method, as the graph distance between cases can readily accommodate edge weights. This would affect the distance between cases inside the trees, but it would not change the multivariate aspect of PERMANOVA - trees remain embedded inside the *n*-dimensional spaces mentioned above.

Paragraph 2: I found it a bit difficult to believe that this addresses a profound gap in the general graph theory literature, and a bit of searching turned up the Python GTST package for a start. There is existing work outside epidemiology. It’s also hard to see how exactly this would be of use in phylogenetic trees; there are developed methods for that and I cannot see the new insight this brings.

Thank you for bringing this paper to our attention, indeed the GTST approach is similar to the PERMANOVA ; the advantage of our approach for the analysis of epidemic forests is that it does not require to choose between different graph kernels but instead uses a metric that makes epidemiological sense, *i.e.* characterising the distance between pairs of cases as the number of generations of infection between them. We are now referring to the GTST paper in the main text line [287]. We have briefly attempted to apply GTST to our simulated data but obtained poor results. A comprehensive comparison of our approaches and GTST’s would require deeper investigations of suitable kernels, and is beyond the scope of the present paper.

Page 10:

Regarding the methods generally: I see no immediate reason why the assumptions underlying these procedures are not met (e.g. PERMANOVA has no assumptions of independence, which is good because trees would strongly violate them) but I think this should be spelled out.

The chi-squared test has assumptions on sample sizes, but we use the non-parametric Monte-Carlo version acknowledged on line 350. PERMANOVA makes no model assumptions and does not require independence, acknowledged on line 359.
Page 11:

Paragraph 3: “Patristic distance” is the wrong term. It is so named because of notions of shared ancestry (and even this was perhaps eccentric - look up “patristic” in the dictionary!) and that does not really make sense here. This is just bog standard graph distance.
Call it graph distance

Thank you, we have renamed it to graph distance.

Paragraph 4: Typo (n-1)/2 should be n(n-1)/2
Thank you.

P12

Paragraph 5: Typo “numbe”

Thank you.

We thank the reviewer for their thoughtful and constructive assessment of our work. We fully acknowledge the concerns regarding the potential arbitrariness in vertex assignment and the extent to which our tests capture meaningful differences between epidemic forests.

In response, we have completely redesigned our simulation framework to ensure that all forests compared share the same set of vertices. We first generate a “reference tree” using a branching process model. From this reference tree, we generate collections of “alternative” transmission trees (epidemic forests) conditional on the reference’s vertex identifiers and infection times, resampling edges according to an offspring distribution parameterised by mean R0 and dispersion parameter k. Forests stemming from the same reference tree but generated with varying offspring distributions are then compared to one another. We have revised the Methods section (“Simulation study” subsection) and the diagram (Figure 4) accordingly.

The simulation study now spans a broad range of generative models, evaluating each test’s performance across combinations of R0 (1.5, 2, 3) and k n(0.1, 0.3, 0.5, 1, Poisson like), resulting in a total of 720,000 forest comparisons.

The chi-squared test is sensitive to node labels, as it compares the frequency of infector-infectee pairs per forest. For PERMANOVA, sensitivity to node labels depends on the chosen distance metric. In our analysis, we use the patristic distance (now renamed standard graph distance), which counts the number of intermediate nodes between any two nodes. The resulting graph distance matrix is symmetric and determined entirely by the tree topology. Although permuting node labels changes the order of rows and columns in the graph distance matrix, the Euclidean distance comparison remains unaffected by such permutations, as the Euclidean distance between matrices is invariant to row and column swaps. Therefore, the result of the comparison is unchanged. However, if a different distance metric, such as Kendall's distance (Kendall et al, 2018 DOI: 10.1214/17-STS637), were used (which measures the depth of the most recent common infector for each pair of nodes), vertices’ labels would matter, specifically the label of the first case.

# Reviewer 2:

The paper addresses an interesting problem, namely inference where the parameter is a tree (graph).

The possible space of trees on N distinguishable nodes is huge and some inferential procedure based on various kinds of data puts a probability distribution (a posteriori, usually) on this space.

Various classical inferential problems then arise: what is a confidence set in this space, how to test equality of two distributions, etc... the information that can be obtained about the distribution is usually simulation based...

In the paper, the theoretical groundwork is somewhat missing... For instance, what is an epidemic forest, said to be a collection of plausible trees? Is it a confidence set? What are defining properties of this forest?

The same lack of precision affects the aim of differentiating two epidemic forests.. is this a test about the possible difference of the underlying measures on the tree space?

Already, talking about obtaining samples from an a posteriori distribution through MCMC brings the problem of dependence/approximate independence between the obtained trees...

The authors present two ad hoc methods to compare two epidemic forests. Both methods are based on reasonable functionals of the trees, but no discussion about other possibilities is presented.

A detailed critique of this spread reconstruction is the small role given in the paper to multiple introductions and missing cases, which are real problems, but avoided in this analysis of the proposed methods. To be honest, this may be a problem also of the models generating the trees that may not allow such complications...

In conclusion, the paper presents a useful ad hoc addition to methods that can be applied to inference yielding trees, but personally I would have appreciated a little bit more theoretical analysis of the underlying inferential problem…

We thank the reviewer for these thoughtful and constructive comments. We agree that our original manuscript lacked sufficient theoretical precision.

**Definition of epidemic forest:** An epidemic forest is a collection of m transmission trees sampled from a probability distribution on the space of labelled trees with ε vertices. This distribution may represent a posterior distribution obtained through Bayesian inference (e.g., via MCMC) or from a stochastic transmission model (added line [337]). The reviewer correctly notes that MCMC-generated trees exhibit temporal dependence. However, our focus is on how to compare and distinguish collections of transmission trees, regardless of how they were inferred. The methods we evaluate (PERMANOVA and χ² test) are permutation-based and do not make any model assumption (added line [369]).

**Simulation redesign:** In response to the requests from all reviewers, we have completely redesigned the simulation study. All forests that are compared to one another now share an identical set of vertices and infection times. We generate a “reference” transmission tree using a branching process model and draw alternative trees (epidemic forest) that retain the same vertices and dates of infections but resample edges under new offspring distributions defined by R0 and k. This redesign ensures that comparisons isolate differences in transmission dynamics rather than differences in vertex labelling, and directly addresses the inferential question posed by the reviewer.

**Comparison to existing methods:** We now acknowledge the GTST approach (Gudmundarson, 2024; DOI: 10.5334/jors.478), which is similar to PERMANOVA. The advantage of our approach for the analysis of epidemic forests is that it does not require choosing between different graph kernels but instead uses a metric that makes epidemiological sense: characterising the distance between pairs of cases as the number of generations of infection between them. We refer to the GTST paper in the main text at line 287.

**Multiple introductions and missing cases:** We agree these are important features of real epidemics. Our current framework assumes single-source outbreaks with complete sampling, which limits immediate applicability but allows us to isolate the core statistical question: can we detect differences in transmission dynamics? Extending the framework to incorporate multiple introductions and unsampled cases would pertain to developing suitable distance metrics for PERMANOVA and would be better suited to a dedicated study.

—

# Reviewer 3:

Reviewer #3: This manuscript addresses the challenge of developing an approach to compare ensembles of transmission trees, which the authors call ‘epidemic forests’. This problem arises naturally in comparing outputs from Bayesian packages for transmission tree reconstruction, though the authors later argue that the methods could have broader application to phylogenetics or other fields. They propose two methods, one based on a chi-square test and one based on PERMANOVA, and evaluate the performance of the tests on simulated data. In particular, they constructed pairs of forests that differed by a tunable amount, and tested the sensitivity and specificity of the two methods to correctly discern the difference across a range of forest sizes and tree sizes. They conclude that the PERMANOVA approach is superior across almost all circumstances, except for quite small forests and relatively small tree sizes where the chi-square approach is better. They make these methods available in an R package.

I found the manuscript very clearly written, with concise text and clean lines of logic throughout. The problem is described and motivated well and the analysis and results are mostly very well explained. The manuscript is quite brief but conveys the concepts well and draws a fairly clear conclusion. I think these will be useful tools for the community working on MCMC reconstruction of transmission trees, and possibly for other communities as well.

However, the brevity of the manuscript also leads to shortcomings, in terms of how convincing the analyses are, and how thoroughly they are discussed. I did not feel that the broad and confident conclusions were supported by the evidence presented, and was not convinced by a key aspect of the methods. I summarize my concerns below.

1. The largest issue is that the authors draw broad conclusions about the relative performance of the two methods, but this is based on a quite limited analysis of simulated data that differ in a single dimension. The simulation leads to two ‘source’ forests G_Pois and G_NegBin, which are generated by a two-step process of simulating two original trees under very different assumptions about the offspring distribution, and then generating a forest from each of them by attempting reconstruction using a separate R package, outbreaker2. Pairs of ‘test’ forests are then drawn from these two source forests – one as a straight subsample (with replacement) from G_NegBin, and the other as a weighted mixture of draws from G_Pois and G_NegBin. The weighting parameter (called the overlap frequency) is then a measure of how similar the two test forests are, which enables the authors to assess sensitivity and specificity of their forest comparison methods. There are several issues here:

a) The mixed forest arising from this construction is a curious hybrid, comprised of a discrete mixture of two very different types of tree. While such a mixture could arise (e.g. if an MCMC chain is hopping between two distinct solutions in tree-space), it is certainly not a general model for two forests that differ from each other – especially since one of the ‘types of tree’ is drawn from the same distribution as the forest we are comparing to. To be blunt, this feels like a peculiar special case contrived to yield a clean series of comparisons, but likely not very relevant to real-world comparisons.

b) A more natural comparison would be between two forests that drift continuously away from each other in tree space (i.e. analogous to two unimodal distributions, rather than a unimodal vs a bimodal mixture distribution). This could be accomplished in the current context by constructing a series of forests where the negative binomial k takes a series of values, e.g. 0.1, 0.3, 1, 3, Poisson. Using this approach, the authors could still generate a series of comparisons that range from ‘no difference’ to ‘k=0.1 vs Poisson’, but the distributions of trees within each forest would be more coherent. I suspect this would be more relevant to most forest comparison problems that arise in research – or in any case, it is a different test to the mixture distribution in the current ms and would test the generality of the conclusions.

c) Even so, the comparisons in the study would be along a single dimension (k) for fixed values of other parameters (notably R). It would bolster the generality of the conclusions to test another dimension, either by replicating the k series for a few different R values, or by exploring an R series instead.

d) If the authors believe the current series based on the mixture distribution and overlap parameter is valid, then another route toward generalization would be to show results for that and a parameter series.

We sincerely thank the reviewer for their suggestion and have completely redesigned the simulation study to address the points above. We fully agree that the original mixture-based approach with an overlap parameter represented an artificial construction unlikely to reflect realistic forest comparisons.

In the revised manuscript, we have implemented a simulation framework that generates forests varying in their offspring distributions parameters (R0 and k). First, we generate a “reference” transmission tree using a branching process model, recording the set of vertex identifiers and their infection times. Then, we construct multiple epidemic forests by repeatedly resampling edges conditional on the reference tree's vertices and infection times, but under different offspring distributions parameterised by R0 and k. The simulation study now spans a broad range of generative models, evaluating each test’s performance across combinations of R0 (1.5, 2, 3) and k (0.1, 0.3, 0.5, 1, Poisson like) for each epidemic size (ε = 20, 50, 100, 200 cases) totalling of 720,000 forest comparisons. This is explained in our revised methods section.

2. The current ms draws the strong conclusion that PERMANOVA outperforms the chi-square test in almost all contexts, and states some numerical thresholds for epidemic sizes and forest sizes where this statement holds true. This is valid for the results shown, but there is no attempt to test the conclusions (or thresholds) more generally, and no caveats are stated. I recognize that there are limits of space and complexity in how many results can be shown, but either the results should be tested soundly for generality or clear caveats need to be added at appropriate points throughout the ms.

In light of our revised simulation framework, we have clearly highlighted the limitations and conditions under which PERMANOVA outperforms the chi-square test (Results section 3rd paragraph).

3. In the current simulation scheme, differing degrees of ‘contact tracing information’ are provided to the outbreaker2 runs for the negative binomial and Poisson cases. The authors state that this was tuned empirically to achieve reconstruction accuracy from 60-80% across epidemic sizes. This initially seemed reasonable, but upon reflection I realized that this is a hidden ‘fudge factor’ that will shape the quantitative results of the method comparison, since it will shape the ‘within-forest variance’ in tree structure which has direct bearing on the PERMANOVA method. How would results differ if the authors had chosen 50% reconstruction accuracy instead? Or 90%?

We thank the reviewer for this important observation. In our revised simulation framework, we no longer rely on tuning reconstruction accuracy with outbreaker2. Instead, we generate multiple epidemic forests directly from a branching process model by varying the offspring distribution through both the reproduction number (R0) and dispersion parameter (k). This approach ensures that forest entropy, i.e. the variation in infector-infectee relationships across trees within a forest, arises naturally from the transmission dynamics rather than being imposed artificially. Larger outbreaks provide more candidate infectors per case, and highly overdispersed outbreaks produce greater variation in infector–infectee pairs. We quantify this variation across all simulation scenarios (Table \ref{tab:lm-entropy}, Supplementary Fig. \ref{fig:entropy}), covering entropy levels in the 50–90% range, comparable to those observed in real outbreak reconstruction studies. Notably, the two methods respond differently to increasing entropy: the chi-squared test loses power as contingency tables become sparse, whereas PERMANOVA gains sensitivity as additional transmission events reduce variance in within-group distances, increasing the F-statistic. We have added this explanation to the Discussion and expanded the Supplementary Materials to document entropy distributions across all parameter combinations.

4. In the expression for the chi-squared statistic (equation 2), I was not clear how the denominator was chosen. The classical chi-squared statistic has terms (O – E)^2/ E. While I can see that neither of the c_ij parameters is the ‘Expected’ value, they will have roughly equal magnitude and so the statistic as written seems roughly equivalent to adding a factor 2 to the denominator. My clever graduate student (who co-reviewed this ms with me) then came up with a derivation where the factor of 2 canceled out, which may explain the form presented in the ms, but in any case some more explanation is needed.

We thank the reviewer for this comment. The chi-squared formula presented in Equation 2 is equivalent to the classical test if one assumes the two forests have equal size, because the expected count for each infector–infectee pair can be taken as the average across the two forests. We have added this explanation directly below Equation 2 in the Methods.

5. The simulation approach for the branching processes is unconventional, but should be roughly correct if S(0) is much greater than the maximum outbreak size. But the value of S(0) is not stated. (I’m not too concerned about this, since the authors’ approach does not hinge on perfect branching process simulation – it just needs a systematic way to generate different trees. But since it is all posed in branching process terms, it would be good to be accurate to avoid confusing readers.)

We thank the reviewer for this comment. To clarify, we initialise each simulation with 10,000 susceptibles and terminate it once exactly ε cases are reached, thereby avoiding saturation effects. This explanation has been added to the Methods.

6. The abstract makes a bold claim that both methods have ‘near-perfect sensitivity’, but then in the Results it becomes clear that this is true only when the overlap parameter is less than 0.6. This is a very important detail – sensitivity is perfect IF the forests are sufficiently different.

Thank you, we have modified the abstract with the new results.

7. In the discussion, the authors state that the methods can be applied more generally to any problem of comparing collections of graphs. Shouldn’t there be a restriction to acyclic graphs? There is no unique way to compute patristic distances on a cyclic graph. Or if the authors are envisioning a different approach to generating the distance matrices, then please clarify.

We thank the reviewer for this point. Our discussion encourages the use of alternative distance functions depending on the research question or dynamics of interest. For transmission trees, which are inherently acyclic, patristic distances are well-defined; for general graphs that may contain cycles, alternative distance metrics would need to be chosen.

8. Some of the color choices in Figure 1 could be more d

We have used shaper colours.
