# Supplementary material for: A statistical framework for comparing epidemic forests
Source: PLoS Comput Biol. 2026 Jun 1;22(6):e1014271. doi: 10.1371/journal.pcbi.1014271 (PMC13225645; doi:10.1371/journal.pcbi.1014271)
Supplement: Attachment — Submitted filename: response_to_reviewers.docx [file pcbi.1014271.s012.docx]

**Response to Reviewers — Round 2**

April 2026

*A statistical framework for comparing epidemic forests*

We thank both reviewers and the editor for their thoughtful feedback. Below, we address each comment in turn.

**Editor:
1) Thank you for including the following in your data availability statement: 'The resulting data is stored on a Zenodo archive: 10.5281/zenodo.17704455.' However, the accession number provided is currently inaccessible. Please verify and provide an active link to facilitate access to the data.**
Apologies for the issue, we have added the direct URL. Please let us know if you have issues accessing the Zenodo archive.

# Reviewer #1

**I thank the authors for the revisions. The new version begs considerably fewer questions and answers my concerns well for the most part.**

We thank the reviewer for their positive assessment and for the constructive comments that have helped improve the manuscript.

**One outstanding matter is that text S1 really just asserts that the offspring distribution remains the same once the parents are permuted. I would like to see this demonstrated, either in simulations or analytically.**

## Thank you for your suggestion. We have expanded Text S1 with a dedicated simulation study that demonstrates the offspring distribution is preserved under the forest generation procedure. Across nine parameter combinations (R ∈ {1.5, 2, 3}, k ∈ {0.1, 0.5, 3}), we simulated 50 reference trees and generated epidemic forests of 50 trees each, applying right-censoring to account for incomplete follow-up. We now include four supplementary figures: the empirical versus theoretical offspring distribution (Fig. S8), the effect of right-censoring on effective sample size (Fig. S9), a quantile–quantile comparison between forest and reference offspring distributions (Fig. S10), and a deviation plot confirming no systematic bias (Fig. S11). Together, these results demonstrate that the forest generation procedure faithfully preserves the target offspring distribution.

## Minor points

**L123: Typo "test tests".**

This is not a typo: “The *χ² test* tests for differences in the frequency of infector-infectee pairs…”

**L176: Perhaps elaborate on "assigning them to a dedicated category in the edge list". Do you mean you just pre-specify which introduction each case belongs to?**

We have clarified this sentence line 175: “In contrast, the χ2 test can handle transmission trees that have multiple introductions by assigning them specific identifiers e.g. ‘Introduction A’, ‘Introduction B’ etc.

**L260: It's previously been claimed that it's easy to expand to different forest sizes but then it's just said that equal sizes are assumed here.**

When forest sizes are equal (mA=mB), the expected count for each pair simplifies to the pooled average, yielding Equation 2. When forest sizes differ (mA≠mB), we use the standard chi-squared formulation where expected counts are proportional to forest size. We added a sentence to clarify L265.

**L299: "Forest labels" needs a bit of elaboration.**

We replaced “labels” with identifiers for clarity and gave an example line 302.

**The ordering of supplementary files seems off.**

We have reviewed and corrected the ordering of all supplementary files to ensure they are referenced sequentially in the main text.

**Text S1, L38: Typo 'idenitifers'.**

Corrected to "identifiers". Thank you.

**Text S1: I'm a bit leery of the "individual reproduction number" terminology; the same wording has definitely been used for something else in the past (e.g. Fraser, PLOS ONE, 2007), and also I'm not really sure that the idea of a reproduction number attached to one person is coherent.**

Thank you. We have clarified that sentence with: “we sampled Ri ∼ NegBin(R′0, k′) for all ε cases, where Ri denotes the number of secondary infections generated by case I”

**Table S2 does not compile in LaTeX.**

The file complies in Overleaf, we will work with PLOS CB should they have any issues compiling it.

# Reviewer #2

**Reviewer #2: After considering the other reviews, I understand that heuristic methods in tree (or forest of trees) comparison are of interest per se. I (Rev 2) interpreted the paper as essentially comparing outputs of statistical modelling of the same data using different inferential models and, in that perspective, I have had and still have doubts.

If a disease is human-to-human transmitted and one-to-one transmission is assumed, there is a "true" transmission tree. Given data on people involved in the transmission, one may try to "estimate" this tree.
Of course, the estimation may encounter various problems, such as undetected individuals, multiple introductions, imprecise infection times, etc..., but could also profit from data about location of individuals or other contact-tracing-like information. If, furthermore, MCMC-like methodology is used, the result will be an a posteriori distribution on the space of trees from which a sample (more or less iid) may be obtained. The distribution will depend on a priori assumptions made, in addition to data. This sample from the a posteriori is one way of obtaining a forest of trees.

If one considers two estimation procedures having different initial assumptions and/or data, the resulting a posteriori distributions should be different, as a rule. Whether this can be seen by comparing two respective samples from the a posteriori distributions (e.g by significance testing of the hypothesis that the two samples come from the same distribution) should then essentially depend on sample size, since one knows beforehand that the distributions are not the same...

In their answer to the previous review (Simulation design), the authors focus on different assumptions regarding offspring distributions defined by R0 and k. From a statistical point of view, one would then incourage the use of goodness-of-fit statistics and selection of best (or credibility set of best) offspring parameters, rather than significance testing between two alternatives...

I thus still think that, from a statistical methodological point of view, the paper does not have a clear aim, but, seen from a more empirical point of view, I have no special objection to the proposal and study of heuristic methods for comparing forests of trees.**

Thank you for your continued engagement. Our framework determines whether two collections of transmission trees are structurally distinguishable. While we present it in the context of Bayesian outbreak reconstruction, where forests represent posterior samples of plausible trees (Duault et al., 2022), the methodology applies more broadly to any setting producing collections of trees, for instance, epidemic simulations on different contact networks sharing the same nodes.

We agree that goodness-of-fit statistics and model selection criteria are valuable when applicable, but they address a different question. Goodness-of-fit statistics assess how well a model fits observed data, but two models may use different data sources or parameterisations, yielding non-comparable likelihoods. Model comparison criteria such as BIC or DIC account for model complexity but generally require the same underlying data, and are not well suited for models of different dimensions or fitted to different data streams. Our test addresses a complementary question: do two forests, regardless of how they were generated, contain structurally distinguishable trees? For instance, two models with different DIC values may nonetheless produce identical forests, in which case our test would correctly conclude no structural difference. Conversely, models with non-comparable likelihoods may produce distinct forests that our test can detect.

The reviewer suggests that, because two different estimation procedures should in principle produce different posteriors, detecting a difference is merely a matter of sample size. In practice, however, one does not know a priori whether the forests differ. Different methods or data sources can yield identical forests, for instance, when added data are uninformative, or when both methods correctly identify the true transmission tree. Our test is designed for exactly these situations, where the answer is not known beforehand.

Moreover, our results in Figure 2 show that increasing sample size (i.e. forest size) does not trivially improve test sensitivity. For the chi-square test, sensitivity for forests differing only in R0 remains low even with increased sample size (Fig. 2, row 2). These results underscore that the relationship between sample size and power depends on both the method and the nature of the underlying difference between forests, warranting the empirical investigation we provide.

We added a clarification in the Introduction (L41, L54) noting that model selection criteria such as DIC and BIC compare model fit but not the resulting tree structures, further motivating the need for our framework.
